# Is Aortic Regurgitation Associated With Long-Term Ergotamine Use?

**DOI:** 10.1016/j.jaccas.2026.107393

**Published:** 2026-03-20

**Authors:** Eiji Koyama, Noriaki Moriyama, Motoki Nagatsuka, Harumi Nakamura, Tohru Asai, Shigeru Saito

**Affiliations:** a Department of Cardiology, Shonan Kamakura General Hospital, Kamakura, Kanagawa, Japan; b Department of Cardiovascular Surgery, Shonan Kamakura General Hospital, Kamakura, Kanagawa, Japan; c Department of Pathology and Diagnostics, Shonan Kamakura General Hospital, Kamakura, Kanagawa, Japan

**Keywords:** aortic regurgitation, case report, drug-induced valvular heart disease, ergotamine, heart failure, migraine

## Abstract

**Background:**

There are various causes of acquired valvular heart disease (VHD), one of which is drug induced.

**Case Summary:**

A 62-year-old woman with long-term excessive use of ergotamine for migraines presented with a 1-month history of dyspnea and was admitted for chronic heart failure due to severe aortic regurgitation. Various medications were initiated in combination with dobutamine, resulting in symptomatic improvement. Under compensated heart failure, elective surgical aortic valve replacement was performed. Pathological examination of the excised valve revealed abnormal leaflet fibrosis, which is not usually observed in naturally degenerated aortic leaflets, suggesting ergotamine-induced valvular changes.

**Discussion:**

This case involved severe aortic regurgitation due to aortic valve fibrosis, possibly caused by long-term ergotamine use, highlighting the need to consider drug-induced etiologies in the differential diagnosis of VHD.

**Take-Home Message:**

The habitual use of ergotamine can cause valvular fibrosis as an adverse effect, potentially leading to VHD.

## Visual Summary

**Figure:**
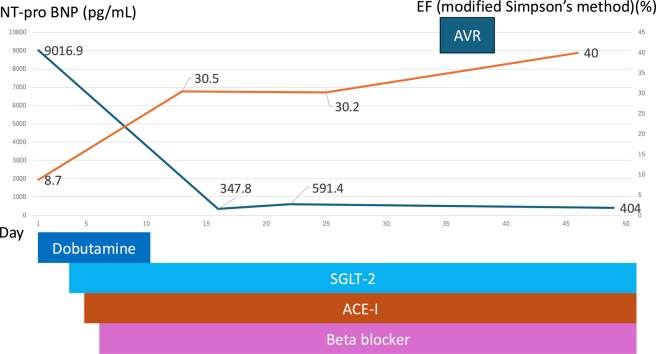

**Visual Summary:**
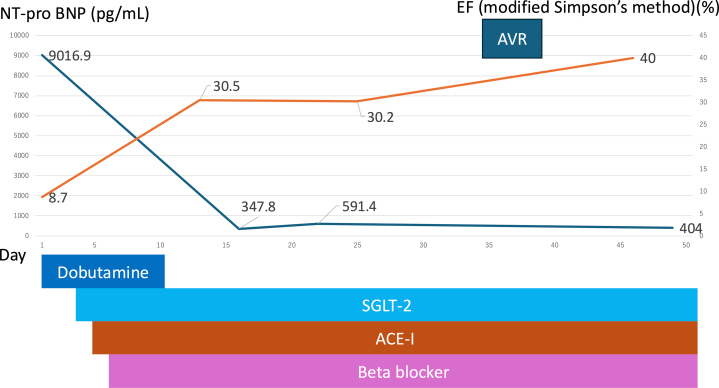
Timeline of Case Presentation AVR = aortic valve replacement; EF = ejection fraction; NT-proBNP = N-terminal pro–B-type natriuretic peptide.

## History of Presentation

A 62-year-old woman presented with a 1-month history of exertional dyspnea, classified as NYHA functional class III. Physical examination revealed a grade 4/6 diastolic murmur and pitting edema in both lower extremities. The remaining examinations were unremarkable.

## Past Medical History

The patient had a history of migraines spanning over 40 years. After unsuccessful trials with various medications for migraines, she ultimately relied on ergotamine for symptom control. She had been taking ergotamine at a dosage of 6 mg/d for 40 years, which exceeded the maximum recommended dosage stated on the package insert. She was also a lifelong smoker.

## Differential Diagnosis

The differential diagnoses for the patient's presentation included ischemic heart disease and both rheumatic and congenital valvular diseases.

## Investigations

**Figure 1:**
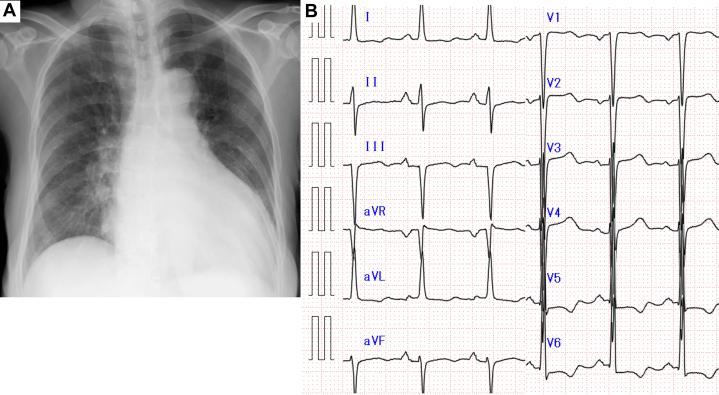
Chest Radiography and Electrocardiography (A) Chest radiography shows cardiomegaly (cardiothoracic ratio: 61.2%) and bilateral pleural effusion. (B) Electrocardiogram demonstrates sinus rhythm at a rate of 94 beats/min, with findings suggestive of left ventricular hypertrophy and left-axis deviation.

**Figure 2:**
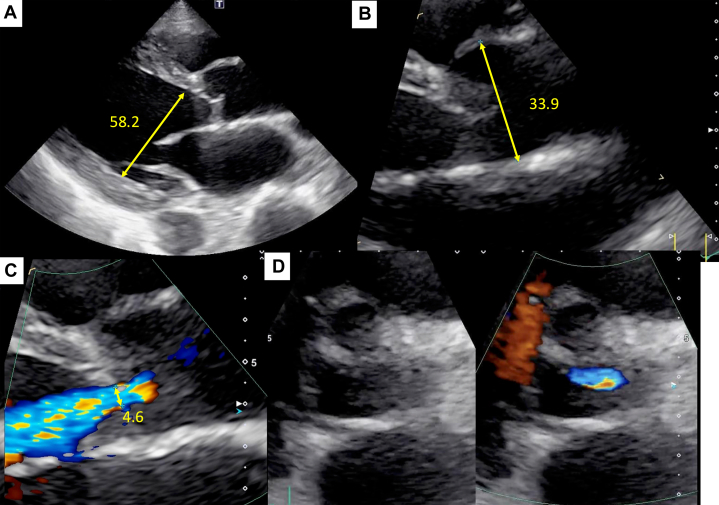
Transthoracic Echocardiography at Admission Transthoracic echocardiography shows moderate-to-severe aortic regurgitation. Left ventricular systolic function was significantly impaired, with an ejection fraction of 21.1% by the Teichholz method and 8.7% by the modified Simpson’s method. The left ventricle is enlarged, with end-diastolic and end-systolic dimensions of 58.2 mm and 52.5 mm, respectively (A, B). The vena contracta width is 4.6 mm (C, D).

Electrocardiography demonstrated a sinus rhythm at a rate of 94 beats/min, with findings suggestive of left ventricular (LV) hypertrophy and left-axis deviation. Chest radiography revealed cardiomegaly (cardiothoracic ratio: 61.2%) and bilateral pleural effusion (Figure 1). Hematological and biochemical tests were within normal limits, except for a markedly elevated N-terminal pro–B-type natriuretic peptide level (9,016.9 pg/mL) (Table 1). Transthoracic echocardiography (TTE) revealed moderate to severe aortic regurgitation (AR), with a regurgitant fraction of 49.9%, regurgitant volume of 20.0 mL, and an effective regurgitant orifice area of 0.12 cm^2^. LV systolic function was significantly impaired, with an ejection fraction (EF) of 21.1% using the Teichholz method and 8.7% using the modified Simpson method. The LV was enlarged, with end-diastolic and end-systolic dimensions of 58.2 and 52.5 mm, respectively. The vena contracta width was 4.6 mm, and the pressure half-time on continuous-wave Doppler was 310 ms (Figure 2, Video 1).

**Table 1:** Laboratory Test Results and Reference Values at Admission

Test	Result	Reference Value
Hemoglobin	14.3 g/dL	11.6-14.8 g/dL
White blood cell count	9.1 × 10^9^/L	3.3-8.6 × 10^9^/L
% Neutrophils	59.00%	41%-71%
% Eosinophils	0.30%	1%-8%
% Lymphocytes	33.00%	19%-48%
% Monocytes	7%	5%-15%
CRP	0.48 mg/dL	<0.14 mg/L
Urea	20.2 mg/dL	8-20 mg/dL
Creatinine	0.83 mg/dL	0.46-0.79 mg/dL
Troponin I	69.04 pg/mL	<45.2 pg/mL
NT-proBNP	9,016.9 pg/mL	<125 pg/mL
Lactate	1.65 mmol/L	0.5-2 mmol/L

CRP = C-reactive protein; NT-proBNP = N-terminal pro–B-type natriuretic peptide.

## Management

After admission, continuous intravenous infusion of dobutamine and intravenous administration of furosemide were initiated. As the patient's symptoms improved, angiotensin-converting enzyme inhibitors, beta-blockers, and sodium glucose cotransporter 2 inhibitors were sequentially introduced.

**Figure 3:**
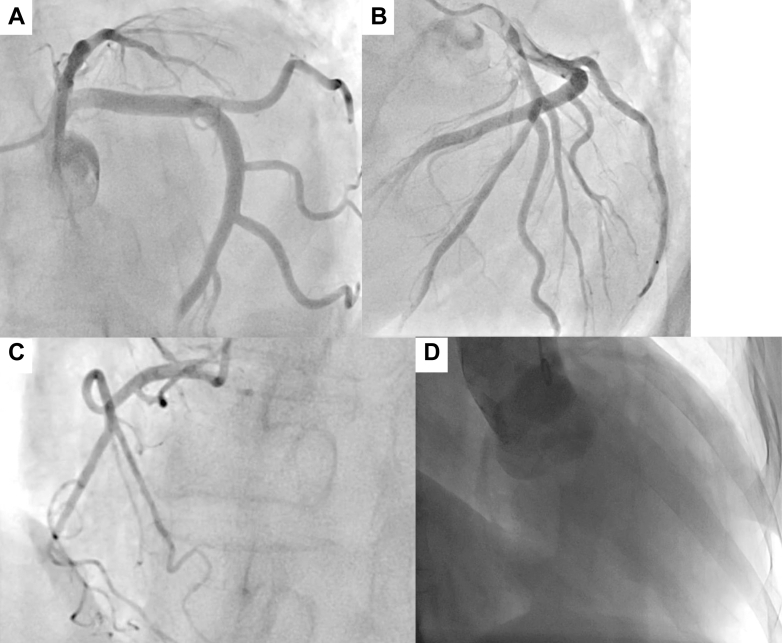
Coronary Angiography and Aortography Coronary angiography shows no significant stenotic lesions (A-C), and aortography reveals Sellers grade 2 aortic regurgitation (D).

**Figure 4:**
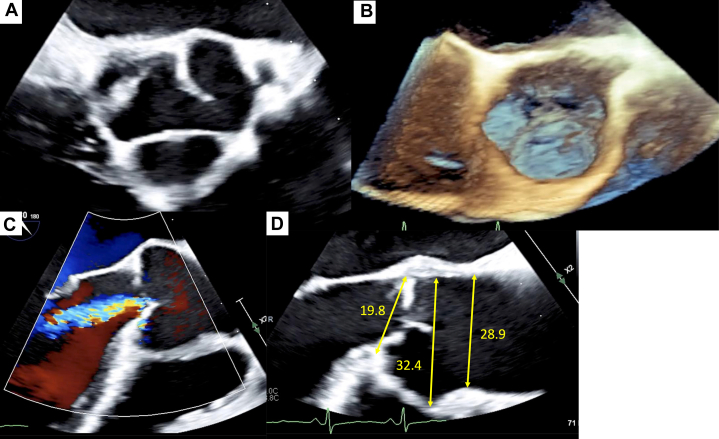
Transesophageal Echocardiography Transesophageal echocardiography shows a tricuspid aortic valve with central regurgitant flow. No dilation is observed in the annulus (19.8 mm), sinus of Valsalva (32.4 mm), or sinotubular junction (28.9 mm). The aortic regurgitation was classified as type 3 (A-D).

Coronary angiography revealed the absence of significant stenotic lesions. Aortography revealed Seller grade 2 AR (Figure 3, Video 2). Transesophageal echocardiography demonstrated a tricuspid aortic valve with central regurgitant flow without leaflet prolapse. No dilation was observed in the annulus (19.8 mm), sinus of Valsalva (32.4 mm), or sinotubular junction (28.9 mm), indicating a normal aortic root other than restricted leaflet motion (Figure 4, Video 3). Therefore, the AR was classified as type 3. Moreover, no significant commissural fusion or leaflet calcification was observed. On follow-up TTE, the EF was 30.2% by the modified Simpson method. Quantitative assessment demonstrated a regurgitant volume of 63.0 mL, an effective regurgitant orifice area of 0.21 cm^2^, and a calculated regurgitant fraction of 56.7%. LV dimensions were essentially unchanged compared with the previous examination, with an end-diastolic diameter of 57.1 mm and an end-systolic diameter of 40.1 mm. Based on these findings, we considered the severity of AR to be moderate to severe.

After multidisciplinary discussion within the heart team, we concluded that the patient had symptomatic moderate-to-severe AR with markedly impaired LV systolic function. Given the clinical presentation and structural valve abnormality, we proceeded with surgical aortic valve replacement after shared decision-making.

## Outcome and Follow-Up

Elective surgical intervention was planned after discharge. On postdischarge day 25, the patient underwent aortic valve replacement via right minithoracotomy with implantation of a 21-mm regent mechanical prosthesis (St Jude Medical).

**Figure 5:**
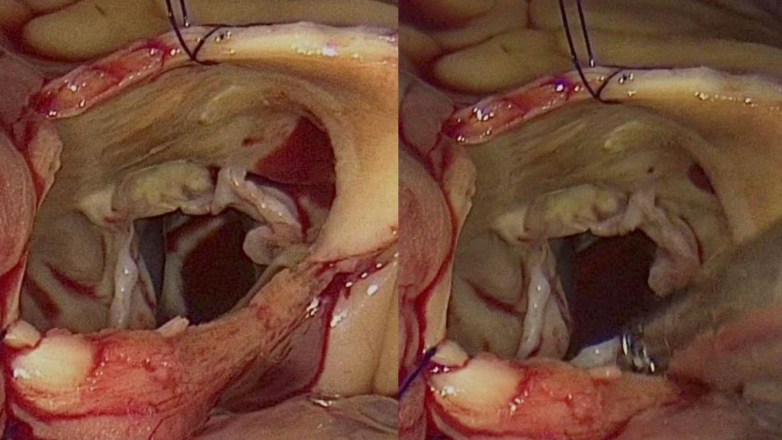
Intraoperative Image of the Aortic Valve Intraoperative findings confirmed a tricuspid aortic valve without gross calcification, perforation, or prolapse. However, all 3 cusps exhibited mild thickening and shortening, resulting in restricted motion and a reduced coaptation area.

Intraoperative findings confirmed a tricuspid aortic valve without gross calcification, perforation, or prolapse. However, all 3 cusps exhibited mild thickening and shortening (Figure 5), resulting in restricted motion and a reduced coaptation area. The postoperative course was uneventful, and the patient was discharged on postoperative day 7.

**Figure 6:**
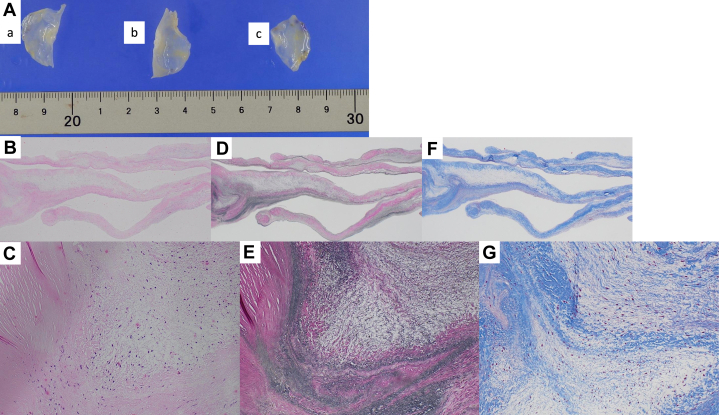
Pathological Examination of the Aortic Valve Pathological examination reveals diffuse thickening and fibrosis of all 3 aortic valve cusps, with minimal evidence of calcification. Notably, the thickening is predominantly localized to the basal portions of the cusps rather than the free edges. Histological analysis demonstrates proliferation of fibroblasts and myofibroblasts, along with nodular areas of fibrosis within the valve tissue. Elastica van Gieson (EVG) staining reveals disorganization of elastic fibers (black), while Masson trichrome (MT) staining demonstrates increased collagen (blue). (A) Macroscopic image of the aortic valve: a) left coronary cusp, b) noncoronary cusp, c) right coronary cusp. (B) Hematoxylin and eosin (HE) staining, 20× magnification; (C) HE staining, 200× magnification; (D) MT staining, 20× magnification; (E) MT staining, 200× magnification; (F) EVG staining, 20× magnification; (G) EVG staining, 200× magnification.

Pathological examination revealed diffuse thickening and fibrosis of all 3 aortic valve leaflets with minimal evidence of calcification. Notably, the thickening was predominantly localized to the basal portions of the cusps rather than to the free edges. Histological analysis demonstrated the proliferation of fibroblasts and myofibroblasts, along with nodular areas of fibrosis within the valve tissue. Immunohistochemical staining of smooth muscle actin (SMA) confirmed the presence of SMA-positive myofibroblasts. These findings are not normally observed in naturally degenerated aortic leaflets (Figure 6). Therefore, it was collectively suggested that the AR may be caused by valvular structural changes induced by prolonged and excessive ergotamine use.

The patient showed a favorable clinical course as an outpatient, with no paravalvular regurgitation on follow-up TTE and an improvement in LVEF to approximately 40%. LV dimensions also showed a decreasing trend, with an end-diastolic diameter of 52.4 mm and an end-systolic diameter of 40.8 mm.

## Discussion

AR results from inadequate coaptation of the 3 aortic valve cusps due to congenital or acquired abnormalities affecting either the valve cusps or the aortic root. The underlying mechanisms include structural changes in the valve cusps themselves, such as rheumatic or age-related degenerative alterations, and aortic root dilatation in the presence of morphologically normal cusps.^1^ In the Framingham Heart Study, the overall prevalence of AR was estimated to be 4.9%, with moderate to severe AR present in 0.5% of participants.^2^ Other studies have reported AR in 13% and 8.5% of men and women, respectively.^3^ The incidence and severity of AR increases with age, peaking between age 40 and 60.^2^ AR can present either acutely or as a chronic valvular disorder, with each form having a distinct etiology, clinical manifestation, management strategy, and natural history.

Drug-induced valvulopathy is a recognized cause of valvular heart disease (VHD). Representative agents include methysergide and ergotamine, which are used for migraine treatment; appetite suppressants, such as fenfluramine and dexfenfluramine; dopamine agonists, such as pergolide and cabergoline; and, more recently, the recreational drug ecstasy (3,4-methylenedioxymethamphetamine), which has also been implicated in valvulopathy.^4^

Migraine is a common neurological disorder affecting up to 6% of men and 17% of women.^5^ Treatment strategies are generally classified into acute therapies aimed at relieving symptoms during attacks and preventive therapies designed to reduce the frequency and severity of recurrent episodes. Methysergide and ergotamine have historically been considered effective agents for migraine prevention. Ergotamine-induced valvulopathy was first reported in 1974,^6^ and cases related to both ergotamine and methysergide have since been documented. Drug-induced valvulopathy most commonly affects the left-sided valves, leading to conditions such as AR and mitral regurgitation,^7^ although tricuspid regurgitation has also been reported.^8^ Despite their limited use today owing to concerns over adverse effects, both methysergide and ergotamine remain approved for migraine-preventive therapy in some settings.

Pathological examination for drug-induced VHD typically reveals glistening white valve leaflets with diffuse, irregular thickening and shortening of the cusps without commissural fusion.^4^ Involvement of the subvalvular apparatus, characterized by shortened and fused chordae tendineae, is also a common finding. Additionally, the proliferation of myofibroblasts and smooth muscle cells occurs within the avascular collagenous matrix surrounding the valve.

In contrast, in cases of AR that progress naturally over time, the aortic valve leaflets become thickened and stiff owing to calcification and fibrosis. In addition, dilatation of the aortic root may occur, accompanied by myxomatous degeneration of the valve cusps.^9^ In rheumatic AR, TTE typically reveals thickening and shortening of cusp edges. Histopathological findings are characterized by fibrotic scarring due to chronic inflammation and neovascularization within the valve tissue.^9^ In the present case, the differential diagnosis included ischemic heart disease and rheumatic or congenital valvular disease. However, the absence of commissural fusion or nodular calcification, the minimal inflammatory scarring, and the lack of aortic root dilatation were less supportive of these etiologies and were more compatible with drug-induced valvulopathy. Nevertheless, causality cannot be definitively established in a single case, and the association should be considered presumptive.

This case involved a patient with a long-term history of high-dose oral ergotamine use for migraine who underwent surgical intervention for AR. The excised valve demonstrated diffuse leaflet thickening with fibrosis without commissural fusion or calcification, findings compatible with drug-associated valvulopathy. However, because alternative acquired etiologies cannot be completely excluded, we describe this case as “possibly ergotamine-associated.” In relatively young patients with AR without congenital structural valve abnormalities or aortic root dilatation, a careful review of the medication history—including exposure to ergot-derived agents—may provide an important diagnostic clue.

## Conclusions

This case highlights the importance of drug-induced etiologies in the differential diagnosis of VHD.

### Data Availability

The datasets used and/or analyzed in the current study are available from the corresponding author upon reasonable request.

## Funding Support and Author Disclosures

The authors have reported that they have no relationships relevant to the contents of this paper to disclose.

**Take-Home Messages**

• The habitual use of ergotamine can cause valvular fibrosis as an adverse effect, potentially leading to valvular heart disease.
• It is important to consider drug-induced etiologies, including those related to ergotamine, in the differential diagnosis of valvular heart disease.

## Appendix

Video 1 Transthoracic Echocardiography at Admission.

Video 2 Coronary Angiography and Aortography.

Video 3 Transesophageal Echocardiography.
